# The Measurement of Reversible Redox Dependent Post-translational Modifications and Their Regulation of Mitochondrial and Skeletal Muscle Function

**DOI:** 10.3389/fphys.2015.00347

**Published:** 2015-11-25

**Authors:** Philip A. Kramer, Jicheng Duan, Wei-Jun Qian, David J. Marcinek

**Affiliations:** ^1^Department of Radiology, University of WashingtonSeattle, WA, USA; ^2^Biological Sciences Division, Pacific Northwest National LaboratoryRichland, WA, USA; ^3^Department of Bioengineering, University of WashingtonSeattle, WA, USA

**Keywords:** redox signaling, post-translational modification, skeletal muscle, mitochondria, myofibrils, glutathionylation

## Abstract

Mitochondrial oxidative stress is a common feature of skeletal myopathies across multiple conditions; however, the mechanism by which it contributes to skeletal muscle dysfunction remains controversial. Oxidative damage to proteins, lipids, and DNA has received the most attention, yet an important role for reversible redox post-translational modifications (PTMs) in pathophysiology is emerging. The possibility that these PTMs can exert dynamic control of muscle function implicates them as a mechanism contributing to skeletal muscle dysfunction in chronic disease. Herein, we discuss the significance of thiol-based redox dependent modifications to mitochondrial, myofibrillar, and excitation-contraction (EC) coupling proteins with an emphasis on how these changes could alter skeletal muscle performance under chronically stressed conditions. A major barrier to a better mechanistic understanding of the role of reversible redox PTMs in muscle function is the technical challenges associated with accurately measuring the changes of site-specific redox PTMs. Here we will critically review current approaches with an emphasis on sample preparation artifacts, quantitation, and specificity. Despite these challenges, the ability to accurately quantify reversible redox PTMs is critical to understanding the mechanisms by which mitochondrial oxidative stress contributes to skeletal muscle dysfunction in chronic diseases.

## Introduction

Oxidative stress plays an important role in skeletal muscle health and disease. In the past, most attention has been focused on skeletal muscle dysfunction caused by oxidative damage to proteins, lipids, and DNA. This damage alters the structure of macromolecules in an irreversible manner and requires turnover and production of new macromolecules to reverse the effects. However, there is growing appreciation of the role of reactive oxygen and nitrogen species (ROS/RNS) in reversible redox post-translational modifications (PTMs) in both muscle adaptation and the effects of chronic disease and aging on skeletal muscle (Sohal and Orr, 2012). Normal skeletal muscle physiology is characterized by transient periods of elevated ROS production due to contractile activity (Powers et al., 2010). In response to repeated bouts of contraction these transient periods of elevated ROS production play an important role in mediating the adaptive benefits of exercise through activation of redox sensitive transcription factors (Powers and Jackson, 2008). On the opposite extreme, chronic elevation of oxidative stress underlies some of the pathological changes associated with disuse atrophy (Min et al., 2011; Powers et al., 2011).

**Figure 1:**
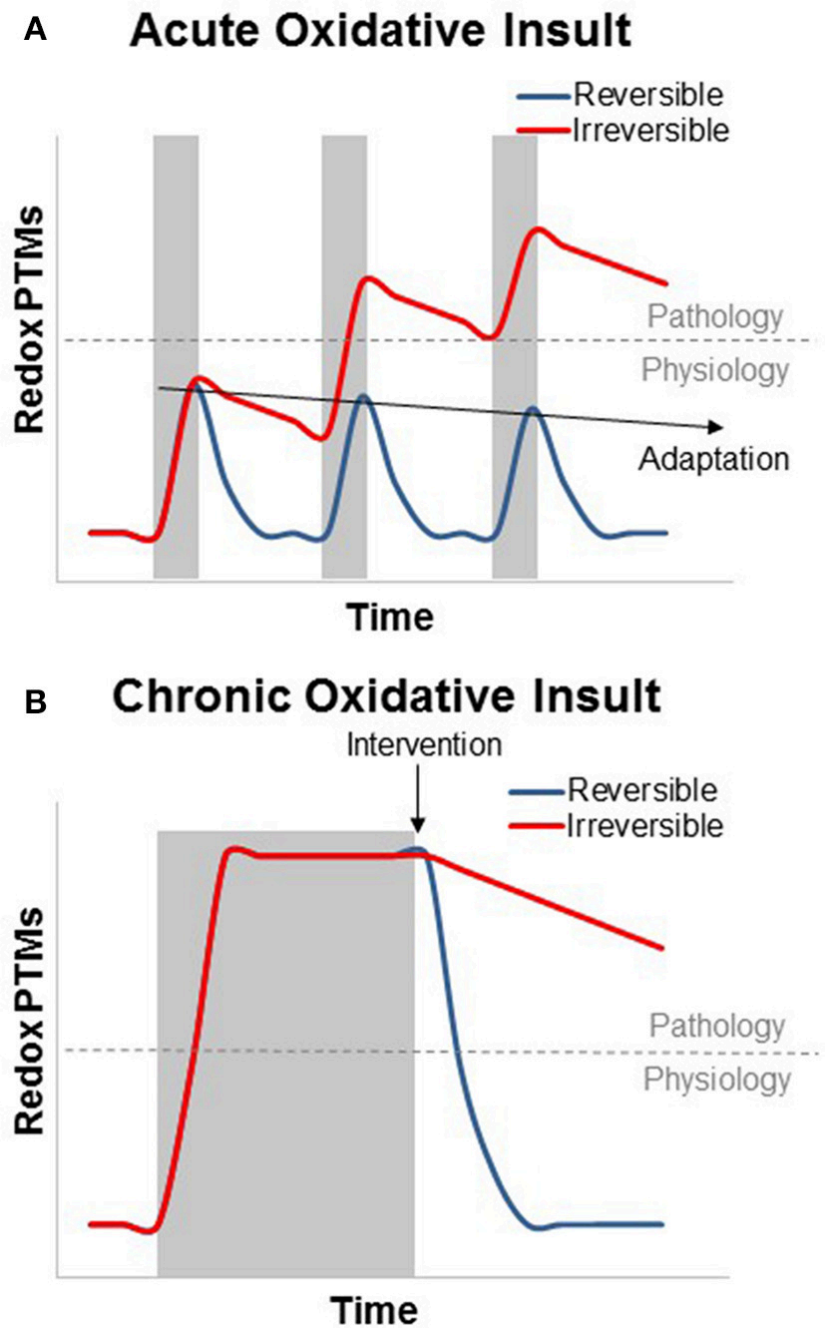
**Theoretical graph of the transient and additive nature of reversible and irreversible redox PTMs in the context of skeletal muscle physiology and disease**. **(A)** Acute oxidative insults (shaded area) can cause a swift and transient increase in reversible oxidative PTMs. Irreversible PTMs rely on protein degradation and turnover, while most reversible modifications can be cleared by the GSH/GSSG and Trx(red)/Trx(ox) redox couples. Subsequent acute oxidative insults can be deleterious if the modifications become irreversible, but this is not the case for reversible modifications, which, can cause transcriptional activation of the antioxidant response (adaptation, preconditioning, or hormesis) to suppress PTM formation. **(B)** Chronic oxidative insults can result in an oxidized redox status which persists throughout the duration of the stress. As with aging, many metabolic and functional skeletal muscle defects that result from this oxidized redox status can be rapidly reversed by restoring a more reduced redox environment. In contrast, irreversible oxidative modifications, however, can persist after the end of the oxidative insult.

In addition to these longer term adaptive changes in muscle, it is now clear that the intracellular redox environment of skeletal muscle also exerts dynamic control over skeletal muscle physiology on the time scale of seconds to hours. Mild elevation of oxidants increases skeletal muscle force production, while further increases in oxidative stress reduce force and play a role in skeletal muscle fatigue (Reid et al., 1993; Andrade et al., 1998; Matuszczak et al., 2005). Manipulation of muscle oxidative stress also induces rapid effects on mitochondrial energetics. Within 24 h of treatment to induce a mild oxidative stress in mice, paraquat impairs *in vivo* mitochondrial energetics and induces an energy stress similar to that observed in aged skeletal muscle (Siegel et al., 2012). Reducing mitochondrial oxidative stress by treating with the mitochondrial targeted peptide SS-31 has the opposite effect. Within 1 h of treatment with SS-31 improved mitochondrial energetics and fatigue resistance were associated with a more reduced GSH/GSSG redox state in aged mouse skeletal muscle (Siegel et al., 2013). The rapid and reversible nature of the control of this activity indicates that these effects are not dependent on turnover of oxidatively damaged molecules or protein translation, but rather are the result of a more dynamic interaction between the redox environment and skeletal muscle physiology (Figure 1), most likely through reversible redox-dependent PTMs. In this review we will highlight key mitochondrial, myofibrillar, and excitation-contraction (EC) coupling proteins whose activities are modified in a redox dependent manner and discuss the challenges and recent developments in strategies to quantify redox PTMs.

## Sources of ROS in skeletal muscle

Skeletal muscle produces ROS from both mitochondrial and non-mitochondrial sources. During muscle contractile activity there is an increase in muscle oxidant production. Mitochondria are frequently cited as the primary source for oxidant production in skeletal muscle. However, growing evidence supports a primary role for non-mitochondrial oxidant by membrane bound NADPH oxidases and, to a lesser extent, extracellular xanthine oxidase production during muscle contraction (Gomez-Cabrera et al., 2005; Frasier et al., 2013; Wadley et al., 2013; Alleman et al., 2014; Sakellariou et al., 2014). In fact, recent work demonstrates that under conditions of increased mitochondrial respiration, as in working muscle, mitochondrial superoxide generation is decreased compared to resting conditions (Anderson and Neufer, 2006; Goncalves et al., 2015). NAD(P)H oxidase enzyme complexes are found in the sarcoplasmic reticulum (SR), the transverse tubules, and plasma membrane. Superoxide produced by SR NAD(P)H oxidases has been implicated in regulation of SR Ca^2+^ dynamics, while plasma membrane NAD(P)H oxidases, along with xanthine oxidase are the main contributors of contraction induced extracellular superoxide production as reviewed by Powers and Jackson (2008). This transient and cyclical contraction induced ROS plays an important role in regulating muscle contraction and inducing adaptive responses to exercise in skeletal (McArdle et al., 2004; Ristow et al., 2009; Powers et al., 2010) and cardiac muscle (Frasier et al., 2013).

In contrast, elevation of superoxide production associated with chronic degenerative disease appears to be primarily mitochondrial in origin. Mitochondrial superoxide or hydrogen peroxide (H_2_O_2_) production is elevated in aging skeletal muscle (Siegel et al., 2013), neurodegenerative disease (Manczak et al., 2011), cardiac pathology (Adlam et al., 2005; Dai et al., 2011a), and following high fat diet. Reducing mitochondrial H_2_O_2_ production with either genetic or pharmacological interventions targeted to the mitochondria reverses or protects against mitochondrial deficits in these conditions (Dai et al., 2011b; Siegel et al., 2013). The mitochondria contain several sites of superoxide production associated with the electron transport system (ETS) and tricarboxylic acid cycle. Complex I of the ETS appears to be the dominant source of mitochondrial superoxide under most conditions with contributions from complex II and III increasing under conditions simulating more active muscle (Goncalves et al., 2015). Complex I and II produce superoxide in the matrix of the mitochondria, while complex III generates superoxide on both sides of the inner mitochondrial membrane (Goncalves et al., 2015). Multiple dehydrogenase enzymes, including pyruvate dehydrogenase and oxoglutarate dehydrogenase, also contribute to superoxide production in the matrix (Mailloux et al., 2014a). Superoxide produced by the mitochondria is rapidly converted to H_2_O_2_ by either MnSOD (matrix) or CuZnSOD (inner membrane space). H_2_O_2_ then interacts with the GSH-thioredoxin redox buffering system or is rapidly decomposed to water and oxygen by the enzyme catalase in the cytosol.

## Redox buffering system

**Figure 2:**
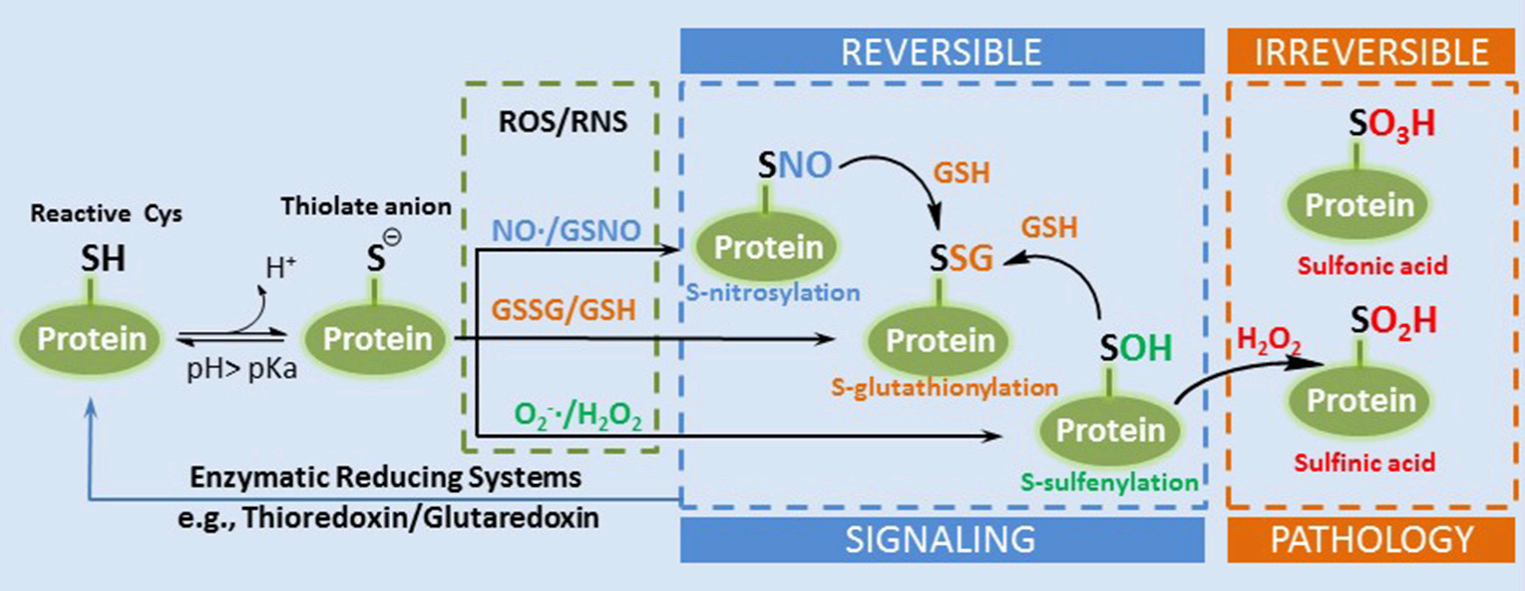
**A simplified diagram of the formation of thiol redox PTMs**. A reactive Cys can exist as thiolate anion under physiological pH. The thioate anion is reactive with different ROS or RNS to form several types of reversible redox PTMs such as SNO, SSG, and SOH. SOH can be further oxidized into irreversible sulfinic and sulfonic acid (Adapted from Filomeni et al. Cell Death and Differentiation 2005, Filomeni et al., 2005).

Cellular redox status, as discussed in this review, is defined as the relationship of ROS producers and scavengers. A common method of determining the redox status of a cell is the reduced to oxidized glutathione ratio (GSH:GSSG). Increasing evidence suggests that redox regulation of cellular processes is the result of signaling rather than non-specific oxidation (Matés et al., 2008). H_2_O_2_, for example, interacts with the redox buffering system through the GSH/GSSG and thioredoxin (Trx_red_/Trx_ox_) redox couples that are present in both the cytosolic and mitochondrial matrix compartments. This redox system, for which NADPH/NADP^+^ provides reducing power, transduces information on the redox status to the cell by interacting with the proteome through the thiol group on cysteine (Cys) residues. Of the greater than 200,000 Cys residues present in the proteome over 20,000 are thought to be redox sensitive, meaning that they can undergo reversible redox modification as described above (Jones and Go, 2011). The susceptibility of the thiol groups on Cys residues, referred to as redox switches, to oxidative modification varies according to their local biochemical environment. The deprotonated thiol, the thiolate anion, is much more susceptible to oxidation than the thiol group. The pKa of thiols is high (>8); however, its proximity to positively charged amino acids in proteins, arginine, and lysine, can function to decrease their pKa values and make them more reactive at physiological pH (Winterbourn and Hampton, 2008). Redox PTMs of thiols are classified as reversible or irreversible, largely depending on the degree of oxidation. The oxidation states of a low pKa thiol (−2), for example, can be readily oxidized to a reversible sulfenic acid (0) by a 2 electron oxidation with H_2_O_2_, and subsequently to sulfinic (+2) and sulfonic acid (+4) which are considered irreversible (Figure 2). These redox PTMs can modify protein function by altering protein structure or modifying interactions with other proteins, ligands, or DNA (Forman et al., 2014).

## Thiol-based redox PTMs

In addition to S-sulfenylation, S-nitrosylation (SNO) and S-glutathionylation (SSG) are two of the most studied reversible redox PTMs (Figure 2). These modifications have been well-recognized as a fundamental mechanism of redox signaling by modulating enzyme activities and protein functions in a variety of cellular activities, such as signaling, metabolism, gene expression, and apoptosis (Janssen-Heininger et al., 2008; Winterbourn and Hampton, 2008; Brandes et al., 2009; Antelmann and Helmann, 2011). The types or levels of PTMs depend on the presence of ROS and RNS, and the redox buffering system, and the enzymatic reduction systems. Collectively, these modifications represent a continuum that relates the form of modification to the extent of ROS/RNS damage and functional consequence. However, our current knowledge of redox PTMs and their significance is still relatively limited due to the technical challenges associated with the measurement of these labile modifications.

## Approaches for measuring reversible redox PTMs

To measure endogenous redox PTMs, one of the major challenges is how to preserve the modifications during sample storage and prevent artificial oxidation during sample preparation. There are two general processes of introducing artifacts: (1) thiol oxidation by air through sample processing; (2) reduction of PTMs by antioxidant enzymes during sample storage or processing (Held and Gibson, 2012). Therefore, special care and sample processing protocols are required in order to accurately measure *in vivo* redox PTMs. To minimize oxidation artifacts during cell or tissue processing, two techniques are commonly applied. These are trichloroacetic acid (TCA) quenching and cell lysis directly with a relative high concentration of alkylation reagents, such as N-ethylmaleimide (NEM) and iodoacetamide (IAA), to block free thiols (Leichert and Jakob, 2006). TCA quenching is a fast process, which can cause precipitation and denaturation of proteins, thus suppressing reactivity of thiolate anions by lowering the pH below their pKa. In contrast, alkylation with NEM and IAM is applied to covalently block free thiols quickly, thus preserve the redox PTMs for downstream processing and measurements. NEM reacts with thiols via a faster and more specific Michael addition reaction than the nucleophilic substitution reaction with IAM. NEM is also less pH dependent compared to IAM, which makes NEM often the preferred blocking reagents for measuring redox PTMs. For measuring redox PTMs in tissues such as muscle, a detailed optimization of tissue handling and processing is particularly important to make sure that the endogenous PTMs are well-preserved during this process.

**Figure 3:**
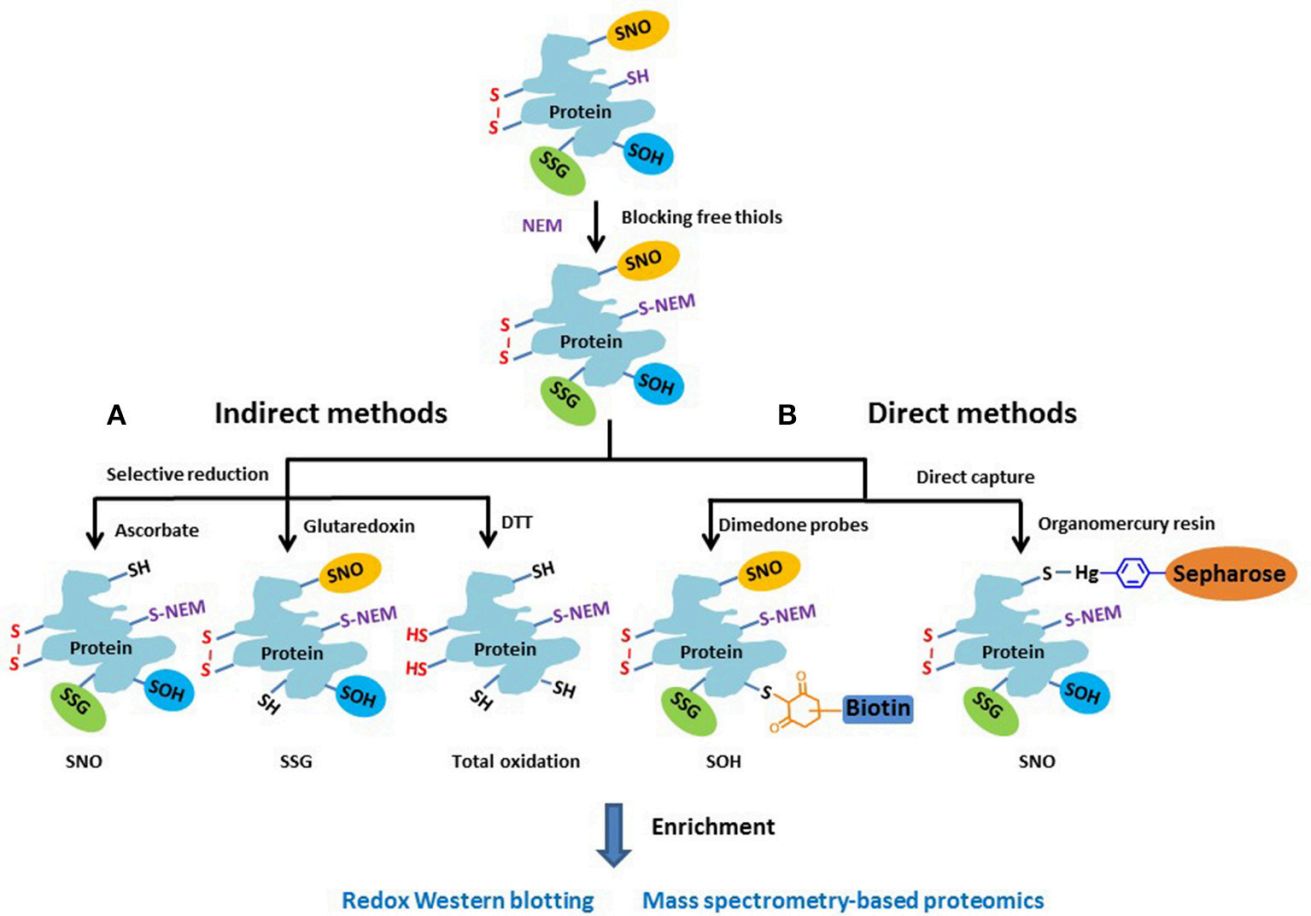
**Schematic of the general approaches for measuring different reversible thiol-based redox PTMs**. The free thiols are typically blocked by alkylation with NEM as the initial step. **(A)** In the indirect methods, the different types of modified cysteines are selectively reduced to free thiols by using individual sets of reagents. **(B)** In the direct methods, dimendone probe is often used to react with SOH, and organomercury resin for SNO. The reduced or labeled protein samples are then subjected to enrichment and measurement by either gel-based or MS-based proteomic approaches.

Approaches for measuring redox PTMs include both indirect and direct detection methods. Given the lack of direct detection approaches of redox PTMs, the indirect approaches are still a popular choice. Figure 3 illustrates the general chemistry principles of most indirect and direct measurement approaches. Basically, the free thiols are initially blocked by alkylation with NEM in a denaturing buffer during tissue homogenization or cell lysis. For indirect methods (Figure 3A), the different types of redox PTMs are selectively reduced to free thiols by using individual sets of reagents where ascorbate is commonly used to reduce SNO (Derakhshan et al., 2007; Su et al., 2013); a glutaredoxin (Grx) reduction cocktail for SSG (Shelton et al., 2005; Zhang et al., 2011), and DTT for total reversible oxidation (Leichert et al., 2008; Paulech et al., 2013). Then, the pre-processed protein samples containing free thiols can be subjected to different enrichment strategies and to either gel-based or mass spectrometry (MS)-based approaches for profiling redox PTM. It should be noted that the selective reduction strategies used in most indirect detection methods are not perfect in terms of specificity. For example, besides reducing SNO, ascorbate is also reported to reduce disulfides in a certain degree (Kuncewicz et al., 2003; Dahm et al., 2006). Similarly, even with the use of mutated form of Grx to reduce its chance for disulfide reduction, non-specific reduction of the Grx enzyme cocktail was still observed (Su et al., 2014). In this regard, the inclusion of proper negative or positive controls when applying these indirect methods is important for more confident identifications of endogenous redox modifications (Guo et al., 2014a).

The concept of indirect detection of redox PTMs was first reported as the biotin-switch technique (BST) for S-nitrosylated proteins (Jaffrey and Snyder, 2001; Jaffrey et al., 2001). This approach was also modified to detect other redox PTMs including SSG (Lind et al., 2002; Reynaert et al., 2006) and total reversible thiol oxidation (Leichert and Jakob, 2004). In this approach, the reduced thiols were labeled with reagents containing a biotin tag and labeled proteins were enriched by avidin affinity purification for biochemical (e.g., Western blotting) analyses. For example, redox differential in gel electrophoresis (redox DIGE) allows the comparison of the levels of oxidation between two samples on the same gel by using iodoaceamido or maleimide-based cyanine fluorophores (Leichert and Jakob, 2004; Hurd et al., 2007).

With the advances of MS-based proteomics a number of liquid chromatography-tandem mass spectrometry (LC-MS/MS)-based proteomics approaches have been developed for identifying and quantifying redox PTMs at the proteome level. For example, in BST the biotinylated peptides (formerly SNO-modified) were enriched by avidin chromatography and the biotin tags were removed to allow the detection of SNO modified Cys sites (Hao et al., 2006). Similarly, the OxICAT approach was developed to measure the levels of total reversible thiol oxidation by labeling the reduced and oxidized thiols with two versions of alkylating ICAT (isotope-coded affinity tags) reagents (Leichert et al., 2008). The differentially labeled thiols with ICAT are purified by biotin-avidin purification and followed by LC-MS/MS to quantify the levels of thiol oxidation on Cys residues based on the light and heavy-labeled peptide intensity ratios. Although the BST or biotin-based approaches are still popular, these approaches generally suffer from the issue of non-specific binding during enrichment. Alternatively, our laboratory developed a resin-assisted capture (RAC) approach (originally called quantitative cysteinyl-peptide enrichment technology, Liu et al., 2004, 2005) for high-efficiency enrichment of cysteine-containing peptides using Thiopropyl Sepharose 6B thiol-affinity resin. More recently, we and others have demonstrated the effectiveness of enriching several types of redox PTMs including SNO, SSG, and total oxidation using this approach (Forrester et al., 2009, 2011; Liu et al., 2010; Paulech et al., 2013; Su et al., 2013, 2014; Guo et al., 2014a,b). Compared to the biotin-based approaches, the resin-assisted procedure provides a simpler workflow, a very high enrichment specificity (>95% of peptides as Cys-peptides), better sensitivity than biotin avidin-based enrichment as demonstrated by a side-by-side comparison (Forrester et al., 2009). The RAC also provides the flexibility for enabling multiplex quantification by allowing on-resin digestion and isobaric labeling. For example, 4-plex (iTRAQ, isobaric tags for relative and absolute quantification), 6-plex and 10-plex TMT (tandem mass tags) can be applied to enable MS-based site-specific identification and quantification of redox PTMs across 4–10 biological conditions (Forrester et al., 2009; Su et al., 2013, 2014; Guo et al., 2014a,b). More recently, a new isobaric reagent iodoTMT that contains an iodoacetyl reactive group was developed to directly label free thiols to enable multiplex quantification of multiple redox PTMs (Pan et al., 2014; Qu et al., 2014). In this approach, the labeled peptides are enriched by an anti-TMT resin. Both the resin-assisted and iodoTMT approach offer the flexibility of multiplexed quantification; however, the resin-assisted approach should provide higher enrichment specificity due to the covalent capture process compared to the non-covalent immunoaffinity enrichment.

Besides the popular indirect methods, several direct measurement strategies (Figure 3B) have been developed for redox PTMs. For example, dimedone-based probes have been shown to specifically label SOH (Charles et al., 2007; Leonard et al., 2009). By incorporating a clickable dimedone probe with a UV-cleavable biotin, MS-based global profiling of SOH modified Cys sites was recently demonstrated (Yang et al., 2014, 2015). For SNO, organomercury resin has recently been applied for direct and site-specific identification of SNO-containing peptides by first blocking the free thiols with MMTS and capturing SNO directly by organomercury resin (Doulias et al., 2010, 2013; Raju et al., 2015). Compared to indirect methods, the direct measurement strategies are less prone to potential artifacts for redox PTMs; however, to date the indirect approaches such as RAC or iodoTMT still provide the most flexibility in multiplexed quantification over many biological samples.

Besides these modifications shown on Figure 3, protein S-sulfhydration (SSH) has recently been reported as a novel thiol-based PTM similarly to SNO (Paul and Snyder, 2012). A modified version of the BST method was applied to detect proteins modified by S-sulfhydration (Mustafa et al., 2009); however, the blocking reagent MMTS (methyl methanethiosulfonate) was shown to react with both free thiols and persulfides (Pan and Carroll, 2013). More recently, a tag-switch assay was reported for more specific SSH detection (Zhang et al., 2014). Further, studies are necessary to validate the specificity and effectiveness of these approaches for SSH modification.

In the study of redox PTMs, it is also important to be able to be quantify the stoichiometry of redox PTMs (i.e., ratios oxidized vs. reduced thiols) in order to better identify functionally important Cys sites. To date, such measurements were only demonstrated by a few approaches such as OxICAT (Leichert et al., 2008; Knoefler et al., 2012) and the RAC approach coupled with TMT (Guo et al., 2014b) in different biological systems. This would be an important area of further development. Furthermore, targeted quantification of redox PTMs by MS is also an interesting development in its ability to perform precise multiplexed quantification of many site-specific PTMs. The proof-of-concept was demonstrated by coupling differential alkylation of free/oxidized thiols using NEM and d5-NEM and multiple reaction monitoring (MRM) to quantify site-specific Cys oxidation status of an endogenous protein p53 (Held et al., 2010). The targeted quantification strategy should have a good potential to complement Western blotting to verify redox PTMs in specific proteins.

## Control of protein structure and function by redox PTMs

Changes in protein structure due to an oxidative environment, can affect the localization, proteasome clearance, and the function or activity of a protein depending on the site, type, and extent of the oxidative modification. The formation of a disulfide bond, for example, can affect the proteins tertiary structure, making it a target for proteases. Many proteases have evolved to target oxidatively modified proteins, thus turning over many of the dysfunctional or structurally abnormal proteins (Jung et al., 2013). Lon protease, for example, has been shown to preferentially target oxidized mitochondrial aconitase in skeletal muscle (Bota et al., 2002). However, heavily oxidized proteins, like aconitase, can form aggregates and become inaccessible to the proteasome (Bota and Davies, 2002). In muscle, creatine kinase has been shown to be oxidative modified in aged mice, resulting in protein aggregates and the loss of enzyme activity (Nuss et al., 2009). Oxidative and other PTMs can have a direct effect on protein function independent of structural changes. Actin, for example, has many reported PTMs that affect its polymerization, organization, and stability (Chung et al., 2013; Terman and Kashina, 2013). These PTMs can form in an enzyme active site with similar functional consequences as will be discussed in this review.

A less recognized role of oxidative PTMs is their ability to regulate the formation of other PTMs associated with normal physiological signaling. This occurs primarily by oxidative modification to a kinase or phosphatase, thus regulating its ability to change the phosphorylation status of a protein. Increasing evidence points to the significance of cross-talk between redox and phosphorylation based signaling systems (Duhé et al., 1998; Filomeni et al., 2005; Chiarugi and Buricchi, 2007; Kemble and Sun, 2009). Indeed, oxidative modifications to the cysteine of protein tyrosine phosphatases has been shown to suppress their activity and lead to the increase in total phosphorylation (Rhee et al., 2000). Intracellular redox circuitry is regarded as a master regulator of phosphorylation and de-phosphorylation because nearly all classes of protein phosphatases and many kinases contain redox sensitive Cys residues for regulating their activities (Chiarugi, 2005; Fisher-Wellman and Neufer, 2012). In general, a shift in cellular redox state such as in aging (Sohal and Orr, 2012) would impact the activities of many phosphatases and stress-sensitive Ser/Thr kinases (e.g., JNK/ASK1, IKKβ, NF-κB), many of which are implicated in insulin resistance (Boura-Halfon and Zick, 2009). Indeed, redox modulation of phosphatase activity and phosphorylation in intact skeletal muscle has been reported (Wright et al., 2009). Moreover, the redox regulation of JNK activation and inactivation of MAP kinase phosphatase (MKP-1) has been reported in cellular aging (Dasgupta et al., 2010).

**Figure 4:**
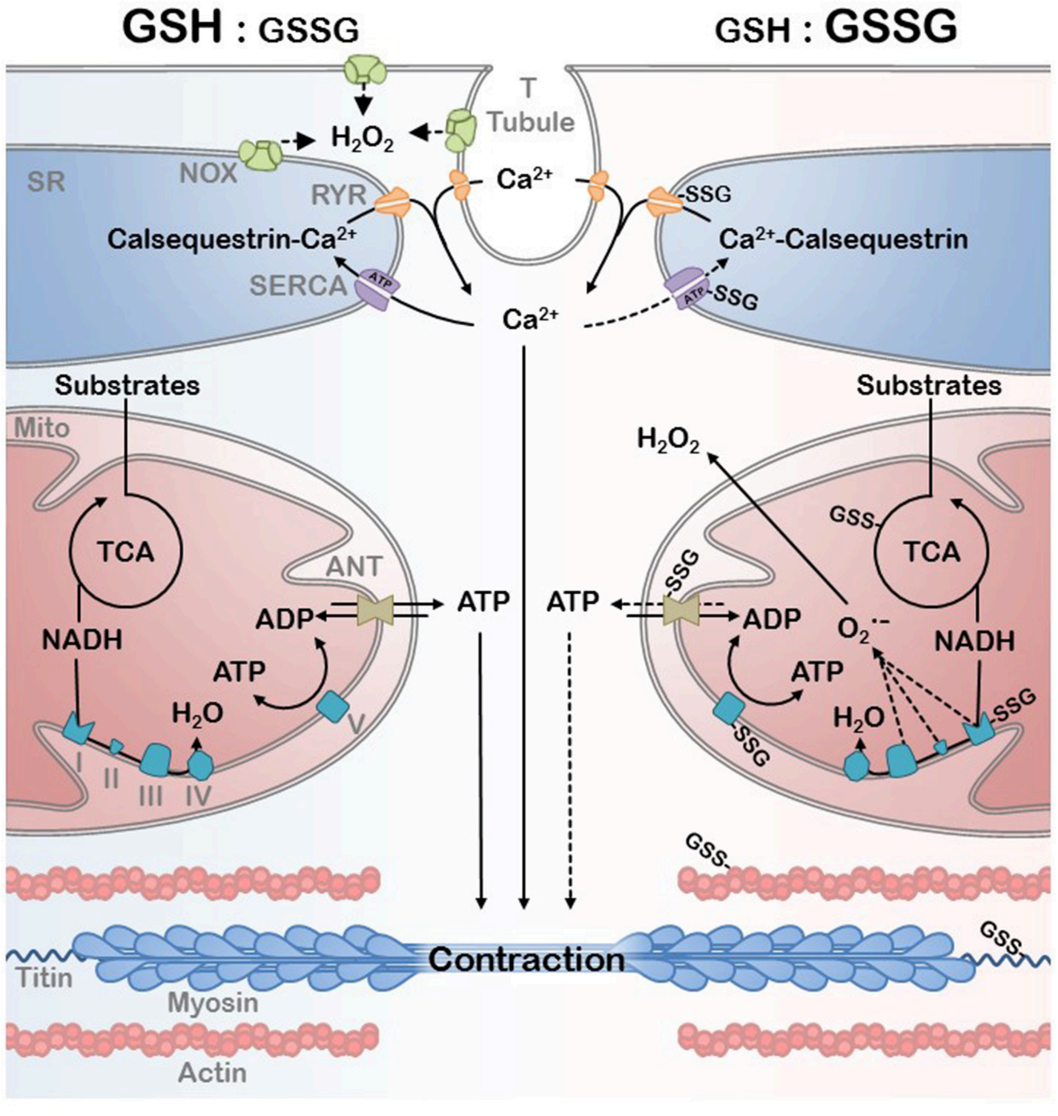
**EC coupling and bioenergetics network in a reduced (high GSH:GSSG ratio) and oxidative (low GSH:GSSG ratio) redox environment**. Ca^2+^ handling by the SR and the ATP generated by the mitochondria are the primary substrates of myofibril contraction. Oxidative PTM can affect the mitochondrial efficiency as well as EC coupling during redox signaling and the oxidative stress associated with disease. Protein S-Glutathioylation of critical mitochondrial, SR, and myofibrillar proteins are depicted as well as the primary sources of H_2_O_2_.

The changes in protein function and their ability to interact with other proteins, macromolecules, and DNA, due to oxidative PTMs is broadly termed redox signaling. However, this is distinguishable from oxidative stress by the degree of oxidative insult, the irreversibility (damage) associated with the PTM, and the specificity of target (Schieber and Chandel, 2014). Aging is an example of a progressive increase in oxidative stress in which physiological redox signaling is disrupted. A redox proteomic analysis has recently demonstrated that the cysteine thiol proteome in aged mouse skeletal muscle is significantly more oxidized than in young adult mice (McDonagh et al., 2014). An important insight from this study was that the number of cysteines that were either reversibly oxidized or available for redox signaling, defined as being partially oxidized, decreased in the aged muscles suggesting reduced flexibility of the redox signaling system in aged muscle. Many mitochondrial and myofibrillar proteins were among the proteins with redox sensitive thiols detected in this study (McDonagh et al., 2014). Below we discuss evidence for the modulation of activity for many of these mitochondrial and myofibrillar proteins by redox PTMs with an emphasis on SSG modification (Figure 4).

## Redox PTMs and mitochondrial function

Mitochondria are attractive targets for redox control. As discussed above, the mitochondria are an important source of superoxide and H_2_O_2_ production in the cell. Therefore, modulation of cell energetics and function by direct control over mitochondrial function by redox PTMs would co-localize the source and targets of oxidant production and allow rapid cellular response to changes in redox status. Mitochondria also contain high concentrations of glutathione and an active thioredoxin (Trx)/GSH redox buffering system that would facilitate translation of oxidative stress to redox PTMs. Finally, many mitochondrial proteins, such as 2-oxoglutarate dehydrogenase, isocitrate dehydrogenase, Complex I, and glutaredoxin isoforms 1 and 2, have conserved cysteine residues associated with dehydrogenase or redox transfer reactions (Mailloux et al., 2014a). Dynamic interaction between mitochondrial respiration and redox biology has been demonstrated in isolated mitochondria, muscle cells, and *in vivo*. Electron flux through the ETS plays an important role in regulating mitochondrial redox status due to the important role of electron flux in controlling NADH/NAD and NADPH/NADP and thus reducing power. An oxidized GSH/GSSG ratio was observed in isolated brain mitochondria under low mitochondrial respiration (rotenone, antimycin A, or no ADP) compared to state 3 respiration (ADP and substrates), however, the GSH/GSSG ratio of freshly isolated mitochondria in the absence of respiratory substrates was more oxidized than either condition (Garcia et al., 2010). The more oxidized GSH/GSSG state was associated with increased protein SSG of several mitochondrial proteins. In a separate study, increases in SSG of mitochondrial proteins reduced basal mitochondrial ATP-linked oxygen consumption as well as mitochondrial reserve capacity in a dose-dependent and reversible manner in smooth muscle cells (Hill et al., 2010). This dynamic interaction between redox status and mitochondrial function also translates to *in vivo* energetics in skeletal muscle. Treatment with paraquat inhibited *in vivo* resting mitochondrial ATPase rate and maximal ATP production within 24 h in aged mice in the absence of changes in mitochondrial protein expression or accumulation of 4-Hydroxynonenal as a marker of oxidative damage (Siegel et al., 2012). Support for an important role of redox regulation of energetics in skeletal muscle pathology also comes from the reversal of mitochondrial deficits in aging muscle following treatment with the mitochondrial targeted peptide SS-31 to reduce mitochondrial oxidant production. One hour after treatment SS-31 increased mitochondrial coupling ratio (P/O) and maximal ATP production (Siegel et al., 2013). This was associated with a more reduced GSH/GSSG ratio in the treated aged muscles. The absence of an observed effect of this treatment in the young muscles or in permeabilized muscle fibers from the treated aged mice suggests that the inhibition of function that was reversed by SS-31 was dependent the interaction between the more oxidized cell environment in the aged muscle and the mitochondria.

Protein SSG of an oxidized cysteine (sulfenic acid), which can be reversed by glutaredoxin, prevents the irreversible oxidation to sulfinic (-SO_2_H) or sulfonic (-SO_3_H) acid. In the case of complex I, irreversible oxidation results in loss of enzyme activity (Hurd et al., 2008). Thus, reversible PTMs can serve as a protective modification during transient oxidative stress. The prevention of further oxidation by SSG was suggested as a mechanism to explain the protective effect of elevated Grx2 expression on doxorubicin-induced cardiotoxicity (Diotte et al., 2009). This study was unique in demonstrating increased SSG of mitochondrial proteins with overexpression of Grx2 under both normal and doxorubicin exposed conditions (Diotte et al., 2009). In contrast, other studies have demonstrated that reduced expression of Grx2 resulted in increased SSG of complex I in the human eye (Liu et al., 2015) and mouse heart (Mailloux et al., 2014b).

In addition to protecting against further oxidation, SSG can also modify protein function. Complex I is one of the most well-studied mitochondrial proteins in terms of redox regulation of activity. The attention given to complex I is likely due in part to its position as the entry point for electrons into the ETS from NADH and its role as a major source of ETS derived superoxide. Therefore, it plays a key role in regulating mitochondrial function and is the complex most commonly implicated in mitochondrial diseases associated with genetic, idiopathic, and lifestyle. Of the 130 cysteine residues contained in the 45 subunits of complex I, 30 were sensitive to reversible oxidation following diamide treatment in mitochondria isolated from brain (Danielson et al., 2011). Six of these residues in brain mitochondria were sensitive to reversible oxidation *in vivo* following depletion of GSH with buthionine sulphoximine (BSO). Two of these residues, cys-531 and 704 on the Ndufs1 subunit were also found to be oxidized in oxidatively stressed bovine hearts (Hurd et al., 2008).

Increased SSG of complex I in heart mitochondria by diamide treatment (Hurd et al., 2008) or by knocking out Grx2 (Mailloux et al., 2014b) leads to reversible inhibition of complex I activity. However, other groups report that the inhibition of complex I activity by SSG was only partially reversible when oxidized under severe oxidizing conditions, suggesting that the SSG may have initiated structural modifications or protein interactions of complex I that were less reversible than the PTM itself (Taylor et al., 2003). Despite the inhibition of complex I NADH reducing activity, SSG of complex I results in elevated superoxide production which is converted to H_2_O_2_ in the intact mitochondria (Taylor et al., 2003). The elevation of superoxide production occurs much more rapidly than inhibition of NADH reducing activity and was acutely reversible with exposure to a reducing agent suggesting that these two effects of SSG on complex I function have different biochemical mechanisms. These functional effects suggest a model whereby an initial disturbance in redox balance could initiate SSG of complex I leading to further oxidation of the mitochondrial environment and inhibition of mitochondrial ATP production, as well as other targets discussed below.

In addition to complex I, the activities of several other proteins involved in oxidative phosphorylation are modified by reversible redox PTMs. F1FO ATPase is another target of redox PTM in the mitochondria. In heart mitochondria disulfide formation and SSG are elevated at Cys 294 on the alpha subunit of the F1 complex, while S-nitrosylation is reduced, in dyssynchronous heart failure (Wang et al., 2011). Cardiac resynchronization therapy that improves cardiac function also reverses the redox PTM at this site. Working with brain and liver mitochondria, Garcia et al demonstrated F1FO ATPase was S-glutathionylated in response to changing mitochondrial GSH/GSSG ratios induced by altering the respiratory state of the mitochondria (Garcia et al., 2010). The increased SSG associated with low respiratory flux reversibly inhibited ATPase activity (Garcia et al., 2010). The supply of reducing equivalents to the respiratory chain can also be modified by redox PTMs. Succinyl CoA transferase (Garcia et al., 2010) and alpha-ketoglutarate dehydrogenase (Applegate et al., 2008) are also reversibly inhibited by SSG in brain and heart mitochondria, respectively. Alpha ketoglutarate dehydrogenase is an NADH generating step in the Kreb's cycle thus inhibition of this enzyme and complex I would reduce mitochondrial capacity by both directly inhibiting the supply of NADH and the entry of NADH into the ETS.

SSG may also exert control over bioenergetics by controlling the coupling of electron transport to ATP generation through the membrane potential. A high potential across the inner mitochondrial membrane under low flux or reductive stress leads to elevated mitochondrial superoxide production (St-Pierre et al., 2002; Nicholls, 2004). Uncoupling proteins 2 and 3 (UCP2 and UCP3) serve as part of the antioxidant defense system by facilitating proton leak across the inner mitochondrial membrane and dissipating the membrane potential to reduce mitochondrial superoxide production (Echtay et al., 2002; Echtay and Brand, 2007). It has been known for several years that oxidative stress induces increased proton leak through UCP3 (Echtay et al., 2002; Echtay and Brand, 2007). Recently Harper and colleagues demonstrated that this proton leak is under the control of the SSG state of Cys 25 and Cys 259 (Mailloux et al., 2011). In contrast to other examples of redox PTM of mitochondrial proteins, where increased SSG is associated with an oxidized GSH/GSSG status, UCP3 appears to be deglutathionylated directly by GRx1 under high oxidative stress conditions (Mailloux et al., 2011). Deglutathionylation of UCP3 led to an increase in state 4 (non-phosphorylating respiration) in mitochondria isolated from skeletal muscle, while treatment with diamide to increase SSG had the opposite effect. Glutathionylation state had a similar effect on the control of UCP2-mediated proton leak in mitochondria from mouse kidney and thymocytes (Mailloux et al., 2011).

Other proton leak pathways may also be regulated by reversible PTMs. The adenine nucleotide transporter (ANT) also plays an important role in regulating mitochondrial ROS production through control of the membrane potential (Brand et al., 2005). There is some evidence that the ANT activity is also affected by SSG (Queiroga et al., 2010), although the evidence for this is not as strong as for other mitochondrial proteins. Modulation of ANT function by redox PTM would provide another route by which the redox status could feedback and control both mitochondrial redox state and energy metabolism of the mitochondria. Thus, SSG of proteins involved in oxidative phosphorylation may represent a dynamic reversible response of the mitochondria to minimize the production of superoxide by the mitochondria or irreversible damage under periods of elevated oxidative stress, such as conditions of low respiratory flux, high reductive stress (high fat feeding), or intermittent exercise (Anderson et al., 2007, 2009). As noted above, SSG can provide a defense against excess mitochondrial superoxide production by modifying the supply of reducing equivalents to the ETS, electron flux through the ETS, and the membrane potential of the mitochondria. However, under conditions of sustained oxidative stress such as chronic disease or aging, these same responses may contribute to mitochondrial energetic deficits and contribute to tissue dysfunction. This suggests that directly altering the redox environment of the mitochondria may be an effective strategy to rapidly improve muscle mitochondrial energetics in the context of chronic disease (Marcinek and Siegel, 2013; Siegel et al., 2013).

It has also been suggested that mitochondrial dynamics, at the level of mitochondrial ultrastructure, are controlled by redox signaling within a cell, which has a direct impact on mitochondrial function (Willems et al., 2015). In C2C12 myocytes, the change in mitochondrial morphology associated with H_2_O_2_ and possibly exercise, is thought to be due to mitochondrial depolarization (Fan et al., 2010), however, increasing evidence of oxidative modifications of mitochondrial fission and fusion proteins (Drp1, Mfn1, Mfn2, and OPA1) suggests redox PTMs may be responsible for regulating mitochondrial dynamics in oxidative environments (Willems et al., 2015). In elderly subjects with a markedly low muscle function, OPA1 protein, mitochondrial respiration and cytochrome c oxidase activity, as well as PGC-1α and Sirt3 protein content were decreased compared to young subjects, suggesting that muscle dysfunction with aging is significantly correlated with changes in mitochondrial function, dynamics, and biogenesis (Joseph et al., 2012).

## Redox PTMs and muscle force generation

The important interaction between ROS, redox status, and muscle force production and fatigue is well-established. Depletion of basal levels of oxidants in resting muscle reduces maximal force, while small elevation of ROS can enhance muscle force production (Reid et al., 1993; Andrade et al., 1998). Several reports have also shown that ROS contribute to muscle fatigue, especially during repetitive submaximal contractions (Reid et al., 1994; Matuszczak et al., 2005). Experimental evidence suggests that ROS may exert their greatest effect on muscle contraction through altering E-C coupling or Ca^2+^ sensitivity of the myofibril (Moopanar and Allen, 2005, 2006), although recent evidence suggests a role for redox PTMs in muscle stiffness (Alegre-Cebollada et al., 2014). Despite the well-established role for redox modulation of muscle contraction, the specific molecular targets for redox modification are less well-defined. Below we discuss recent evidence for effect of redox PTMs on activity of specific E-C coupling and myofibrillar proteins.

Redox modifications of some components of the myofibril appear to be dependent on the contractile state of the protein. This has been hypothesized to be due to structural changes in the proteins that either bury or expose cysteine residues to the cytosolic environment (Gross and Lehman, 2013; Alegre-Cebollada et al., 2014). When cardiac myofibrils were exposed to cysteine oxidizing reagents under rigor, basal myofibrillar ATPase rates were significantly higher than controls, but there was no effect on maximal ATPase activity (Gross and Lehman, 2013). In contrast, myofibrils exposed under relaxing conditions have a significantly lower maximal ATPase activity with no effect on the basal rate. The reduced ATPase rate in the resting condition was associated with increased cysteine oxidation of the myosin heavy chain compared to control and rigor. These effects were similar for different oxidative modifications induced by NEM, SNAP, and DTDP suggesting that the type of modification to the MHC thiols may not be critical for the effect on ATPase activity. Redox modifications of titin also appear to be determined by changes in protein structure (Alegre-Cebollada et al., 2014). Titin is an approximately 3000 kD protein that stretches between the M-line and Z-line and anchors the thick filament of the myofibril to the z-line. It has a highly coiled structure at rest that is stretched during lengthening of striated muscle sarcomeres (Tan et al., 1993). This stretching provides stability and elasticity. Titin has multiple cysteine residues that are buried on the interior of the protein under relaxed conditions, but are exposed during stretching. Alegre-Cebollada et al found that stretching the protein made these cysteines susceptible to SSG which prevented its refolding and modulated the elasticity of cardiac sarcomeres (Alegre-Cebollada et al., 2014). Similarly, increased mechanical stability of titin caused by disulfide bond formation within the cardiac-specific N2-Bus region of the protein is believed to result in the passive stiffness in human heart myofibrils under oxidizing conditions (Grützner et al., 2009). This reduced elasticity of titin by reversible PTM may contribute to impaired relaxation and diastolic dysfunction under conditions of chronic oxidative stress such as aging.

Redox PTMs may affect muscle force production by modulating both Ca^2+^ dynamics and Ca^2+^ sensitivity of the myofiber. Redox proteomic analysis indicates that both alpha and beta isoforms of tropomyosin can undergo reversible redox modification as well as Troponin I, Myosin light chain 1/3, Myosin regulatory light chain 2, and Myosin-4(McDonagh et al., 2014). Following myocardial infarction or ischemia reperfusion in pig, mice, and rats, Cys190, the only cardiac tropomyosin cysteine, showed significant oxidation and was at least partly implicated in the resulting contractile dysfunction by a H_2_O_2_ dependent mechanism (Canton et al., 2004, 2006; Avner et al., 2012).

The effects of redox modulation on Ca^2+^ release from the SR by RyR and reuptake by SERCA may be the most well studied effects of redox PTMs on muscle function. Ca^2+^ reuptake into the SR by the SERCA proteins determines the rate of relaxation of muscle and the ability to sustain repeated muscle contractions. In the mouse heart, SERCA Cys 674 glutathionylation resulted in increased activity; however, irreversible sulfonation resulted in decreased Ca^2+^ reuptake and relaxation and was prevented by catalase overexpression (Tong et al., 2010; Qin et al., 2013). Similarly, treatment of rat ventricular myocytes with H_2_O_2_ resulted in SR Ca^2+^ depletion due to SERCA oxidative inhibition and the oxidative activation of Na+/Ca^2+^-Exchanger (Kuster et al., 2010).

Ca^2+^ release from the SR by RyR is also affected by the oxidation state of its cysteine residues. Stamler et al demonstrated that RyR has multiple cysteine residues that have different levels of susceptibility to oxidation (Sun et al., 2013). Under high pO2 conditions to induce an oxidative stress, cysteine residues in regions of the protein that interact with the L-Type Ca^2+^ channel form disulfide bonds, which may disrupt the interaction of the RyR with this protein. Interaction with the L-type Ca^2+^ channel is responsible for Ca^2+^ induced Ca^2+^ release and disruption of this interaction by thiol oxidation may inhibit full Ca^2+^ release from the SR. Marks and colleagues have also found disruption of the calstabin 1- RyR interaction by both reversible and irreversible PTMs in aged skeletal muscles (Umanskaya et al., 2014) and heart failure (Rullman et al., 2013). This oxidation was prevented by reducing mitochondrial-derived oxidative stress with mitochondrial-targeted catalase expression. Destabilization of the calstabin-1-RyR interaction led to increased open probability of the RyR and greater Ca^2+^ leak from the SR. This chronic leak reduced the Ca^2+^ released from the SR and therefore muscle force production upon muscle stimulation in aging muscle. The chronic Ca^2+^ leak induced by RyR oxidation also induces a feedforward cycle by increasing mitochondrial oxidative stress and muscle atrophy due to increased mitochondrial Ca^2+^ uptake. Both of these effects in aging muscle were reduced in aged mice overexpressing mitochondrial targeted catalase to reduce oxidative stress (Umanskaya et al., 2014).

## Conclusions

Skeletal muscle physiology is determined by EC coupling, myofibrillar force production, and bioenergetics. In this review we have highlighted how each of these processes can be acutely controlled by changes in redox homeostasis through reversible redox sensitive PTMs. These redox PTMs provide a mechanism for cells to rapidly respond to redox changes to prevent irreversible oxidative damage, modulate protein activity or signal adaptive responses to stress. The GSH/GSSG and thioredoxin systems provide the biochemical link between the cellular redox environment and protein thiol groups. Thus, reversible thiol modifications provide a mechanism for cells to rapidly respond to redox changes, which during transient stress can serve important signaling or cell-protective roles by altering protein function. However, under conditions of chronic redox stress that create conditions, where these normally transient modifications persist, these same signaling or protective responses can inhibit cell function and lead to pathology. We have attempted to highlight some potential targets in the skeletal muscle mitochondria, E-C coupling and myofibrillar systems where persistent redox sensitive PTMs may contribute to muscle pathology in chronic diseases and aging. In the mitochondria it is becoming increasingly clear that redox PTMs exert control over both entries of electrons into the ETS as well as flux through the system, thereby dynamically influencing both mitochondrial energetics and oxidative stress. These changes to the mitochondria can then feedforward to influence redox PTMs on E-C coupling and myofibrillar proteins to influence Ca^2+^ sensitivity, relaxation, and force production (Figure 4). Thus, reversible redox PTMs provide a mechanism by which the mitochondria can influence muscle force production that is parallel to, and in some ways, independent of changes in ATP flux. Another important point is that because these changes rapidly respond to changes in the redox environment of the cell they may provide a mechanism to rapidly reverse functional deficits in muscle performance associated chronic disease. We are relatively early in our mechanistic understanding of the contribution of redox PTMs to skeletal muscle dysfunction in chronic disease. As interest continues to grow and technical challenges to the quantitative measurements of these changes *in vivo* are overcome we expect that this area will lead to new insights into the mechanisms of skeletal muscle dysfunction and identify exciting new directions for development of interventions to improve quality of life in this growing patient population.

## Author contributions

All authors contributed to the design, writing, and editing of this review, with PK and DM writing the sections on redox PTMs and skeletal muscle physiology and disease, and WQ and JD writing the sections on methods and technical challenges. PK created Figures 1, 4, and JD and WQ created/modified Figures 2, 3.

## Funding

This work was supported by a Glenn/AFAR Breakthroughs in Gerontology Award and the National Institutes of Health grants P01-AG001751 (DM), P41-GM103493 (WQ), and T32AG000057 (PK).

### Conflict of interest statement

The authors declare that the research was conducted in the absence of any commercial or financial relationships that could be construed as a potential conflict of interest.
